# Influenza D in Domestic and Wild Animals

**DOI:** 10.3390/v15122433

**Published:** 2023-12-15

**Authors:** Malgorzata Kwasnik, Jerzy Rola, Wojciech Rozek

**Affiliations:** Department of Virology, National Veterinary Research Institute, Al. Partyzantow 57, 24-100 Pulawy, Poland; malgorzata.kwasnik@piwet.pulawy.pl (M.K.); jrola@piwet.pulawy.pl (J.R.)

**Keywords:** influenza D virus, IDV, zoonosis, epidemiology, wild animals

## Abstract

Influenza D virus (IDV) infections have been observed in animals worldwide, confirmed through both serological and molecular tests, as well as virus isolation. IDV possesses unique properties that distinguish it from other influenza viruses, primarily attributed to the hemagglutinin-esterase fusion (HEF) surface glycoprotein, which determines the virus’ tropism and wide host range. Cattle are postulated to be the reservoir of IDV, and the virus is identified as one of the causative agents of bovine respiratory disease (BRD) syndrome. Animals associated with humans and susceptible to IDV infection include camels, pigs, small ruminants, and horses. Notably, high seroprevalence towards IDV, apart from cattle, is also observed in camels, potentially constituting a reservoir of the virus. Among wild and captive animals, IDV infections have been confirmed in feral pigs, wild boars, deer, hedgehogs, giraffes, wildebeests, kangaroos, wallabies, and llamas. The transmission potential and host range of IDV may contribute to future viral differentiation. It has been confirmed that influenza D may pose a threat to humans as a zoonosis, with seroprevalence noted in people with professional contact with cattle.

## 1. Introduction

**Figure 1:**
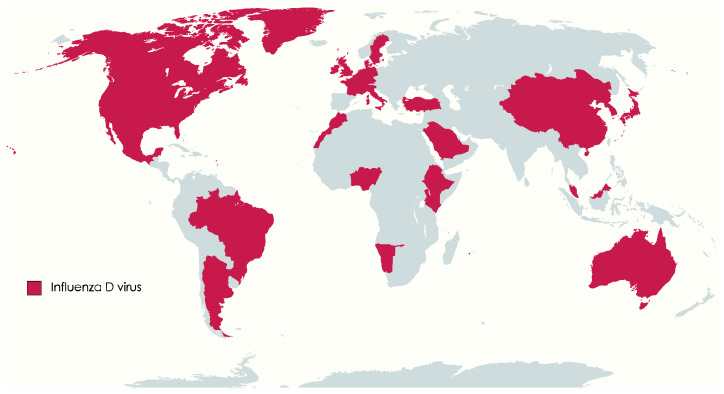
World map: countries where the occurrence of Influenza D has been confirmed molecularly or serologically are marked in purple (Argentina, Australia, Belgium, Benin, Brazil, Canada, China, Denmark, Ethiopia, France, Germany, Ireland, Italy, Japan, Kenya, Luxembourg, Malaysia, Mexico, Mongolia, Morocco, Namibia, Netherlands, Nigeria, Republic of Korea, Saudi Arabia, Sweden, Switzerland, Togo, Turkey, UK, and USA) (map.chart.net accessed on 1 September 2023).

Influenza D virus (IDV) belongs to the *Orthomyxoviridae* family, the genus *Deltainfluenzavirus*, along with influenza viruses of the genera *Alphainfluenzavirus* (Influenzavirus A, IAV), *Betainfluenzavirus* (Influenzavirus B, IBV), and *Gammainfluenzavirus* (Influenzavirus C, ICV). Influenza viruses of distinct types (A, B, C, or D) exhibit variations in genome structure, properties of surface glycoproteins, and the antigenicity of the viral nucleoprotein and matrix protein [1,2]. Influenza viruses belonging to different types also display differing host ranges. Influenza A viruses exhibit broad host tropism, can infect wildfowl, swine, horses, bats, and humans, leading to severe respiratory disease and seasonal epidemics, and have the potential for triggering pandemics [3]. Based on the surface glycoproteins hemagglutinin and neuraminidase, IAV is divided into subtypes (H1 to H18 and N1 to N11). The main hosts for IBV and ICV are humans, IBV may cause seasonal epidemics, while ICV causes only mild illness. IBV has been isolated from seals and pigs [4,5]; antibodies against IBV were also reported in dogs and horses [6,7]. ICV can occasionally infect cattle [8], dogs, and pigs [9,10]. Serological evidence of ICV was reported also in equine populations [11]. Influenza D virus is a relatively recent discovery, first identified in 2011 in pigs in the USA, although evidence indicates its circulation in cattle populations since at least 2003 [2,12]. IDV’s global distribution is now well-established, with reports from Europe, North America, South America, Africa, Asia, and recently Australia [13,14,15,16,17,18] (Figure 1).

Although IDV was initially detected in pigs, cattle are considered the main reservoir of the virus [19]. The increasing number of studies have shown that IDV may be strongly associated with the bovine respiratory disease (BRD) syndrome [20]. The virus has been isolated from both cattle and pigs, while the presence of antibodies for IDV have been identified in various animal species [18,21,22,23,24,25]. Histochemistry on tissue arrays has confirmed IDV’s potential to bind to receptors in the respiratory tracts of various domestic and wild animals [26]. Seroprevalence of IDV has also been established in humans, particularly those in professional contact with cattle [27]. Studies on poultry have not demonstrated sensitivity to IDV infection, but molecular diagnostics have revealed the presence of the IDV’s RNA in aerosol samples from poultry farms in Malaysia [28]. This review provides information on the occurrence of influenza D infections in numerous domestic and wild animal species. Due to its relatively recent discovery, IDV is the least investigated among the influenza viruses. Therefore, we have introduced basic information on the structure of the virion, phylogenetic analysis of circulating strains, disease symptoms, and diagnostic methods.

## 2. Molecular Structure and Receptor Specificity of IDV

**Figure 2:**
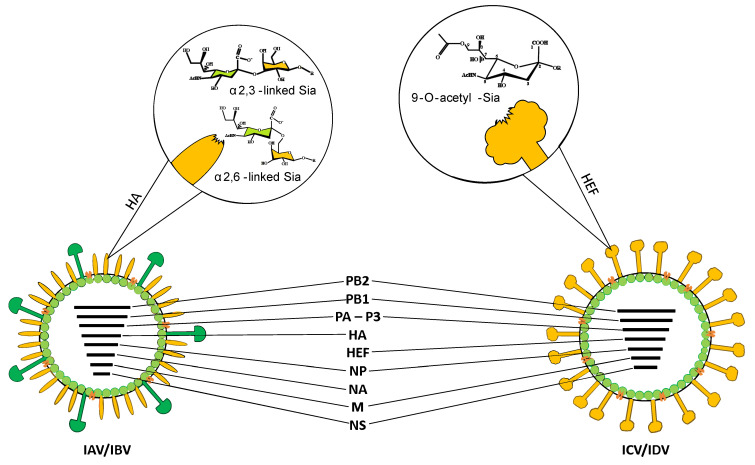
Schematic view of influenza virions. RNA segments are indicated. glycan binding preferences are presented at magnification. HA—hemagglutinin; HEF—hemagglutinin esterase fusion protein.

Influenza virus type D is an enveloped, spherical to pleomorphic virus with a diameter of approximately 100–120 nm, possessing a segmented genome consisting of seven negative-sense, single-stranded RNA segments. The genome organization is akin to that of ICV, differing from IAV and IBV, which have eight RNA segments (Figure 2) [2,29].

Recently, an electron scanning tomography has revealed that some ICV and IDV virions can package eight RNA segments arranged in a specific pattern. Further investigation is required to determine whether the eighth segment comprises duplicated vRNAs or host-derived RNAs, such as ribosomal RNA [30]. Each RNA segment is enclosed in a ribonucleoprotein complex (RNP) containing nucleoprotein and RNA-dependent RNA polymerase. The viral RNA segments encode polymerase proteins PB2, PB1, and P3, hemagglutinin esterase fusion protein (HEF), and nucleoprotein (NP). The M and NS RNA segments undergo alternative splicing. Two proteins responsible for ion channel activity and virion morphology, M2 and M1, as well as two proteins involved in interferon antagonism and nuclear export of RNP, NS1, and NEP, are encoded [31]. The presence of one surface glycoprotein HEF is characteristic of C- and D-type viruses, while type A and B viruses contain two major surface glycoproteins, hemagglutinin (HA) and neuraminidase (NA). Phylogenetic analysis reveals a nucleotide homology of 50% for the HEF segment, the most variable, and 70% for PB1, the most conserved segment, between ICV and IDV [21,32,33].

It appears that the HEF glycoprotein and the M1 and M2 proteins play a crucial role in virus infectivity and determine the virus’s host range. The surface glycoprotein HEF is responsible for initiating virus entry into the cell, binding to receptors, and membranes fusion. It is also the main determinant of the high thermal and acid stability of IDV. Sialic acid residues on the cellular surface glycoproteins act as receptors for influenza viruses [3,34]. IAV and IBV recognize α2,3- or α2,6-linked sialic acid moieties [35]. IDV binds to both Neu5,9Ac2 and Neu5Gc9Ac-containing glycans, irrespective of whether they are attached to galactose via an α-2,3 or α-2,6 linkage (Figure 2). In contrast, ICV exhibits a preference for Neu5,9Ac2 over Neu5Gc9Ac [33,36]. Despite the different expression levels of these two 9-O-acetylated sialic acids in potential hosts, it is postulated that IDV can efficiently bind and spread [33]. Yu et al. compared the stability of IDV with influenza A, B, and C viruses after prolonged incubation at high temperatures or in a low pH environment. Studies demonstrated that treating IDV with a low-pH buffer had no significant effect on virus infectivity, while it completely inactivated IAV [33]. The results suggest that IDV is the most stable among the four types of influenza viruses. It is postulated that pH changes may be important in the activation of the IDV fusion peptide, but this is not a critical factor. In the case of IAV, the relationship between the acid stability of hemagglutinin and the ability to transmit between species was confirmed [37]. Understanding the details of the pH-induced conformational changes of HEF during membrane fusion will likely help to elucidate the host specificity of IDV.

Mechanisms of NS splicing are common among influenza viruses. In contrast, differences in the mechanism of M-segment splicing for ICV and IDV have been observed. In the case of ICV, the M1 gene is produced via splicing, introducing the termination codon into the spliced mRNA. For IDV, an additional 4-amino acid coding sequence is added to the preceding exon during the splicing of the M segment [2]. Studies on RNA splicing of the M segment of IAV have suggested its importance in the context of the virus’s host range [38]. It is possible that the mechanism of M1 and M2 protein synthesis among different types of influenza viruses may determine the viral host range, tissue tropism, and low pH sensitivity.

The study of the general codon usage pattern of the IDV HEF and PB1 genes was conducted to understand the direction of evolutionary changes of this virus. The results confirmed the adaptation of IDV to multiple hosts, especially cattle. Additionally, the similarity index (SiD) analysis revealed that pigs exerted a stronger evolutionary pressure on IDV than cattle. Furthermore, the conserved PB1 gene showed a similar codon usage pattern compared to HEF. The authors postulate that IDV is capable of maintaining infection in multiple hosts [39].

## 3. Phylogenetics

**Figure 3:**
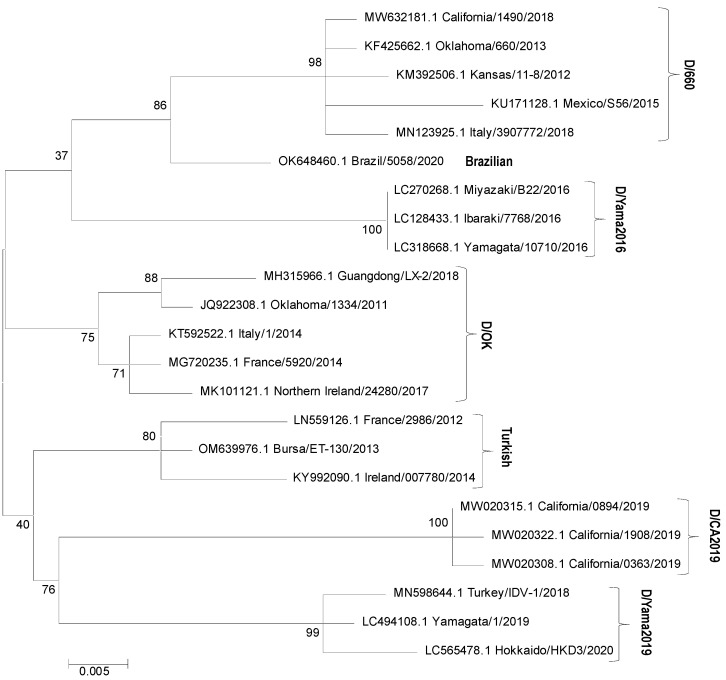
Phylogenetic tree based on nucleotide sequences of IDV HEF segment (674 nt fragments). Maximum likelihood analysis in combination with 1000 bootstrap replicates was used; bootstrap values were given at relevant nodes. IDV HEF sequences available in the GenBank database were included in the analysis; their accession numbers are submitted in the figure.

Phylogenetic analyses have revealed that IDV circulates globally in at least four distinct genetic lineages: D/swine/Oklahoma/1334/2011 (D/OK), detected in many European countries, the USA, Mexico, China, and Namibia; D/bovine/Oklahoma/660/2013 (D/660), identified in Italy, the USA, and Mexico; D/bovine/Yamagata/10710/2016 (D/Yama2016), found in Japan; and D/bovine/Yamagata/1/2019 (D/Yama2019), detected in Japan and China [20,40,41,42,43]. Viral strains from potentially new lineages were identified in California (D/CA2019), Brazil, and Turkey [14,44,45]. Phylogenetic relationships are illustrated in Figure 3. Viral strains from lineages D/OK and D/660 were reported to frequently reassort with each other and exhibit antibody cross-reactivity. However, strains identified in the cattle population in Japan were distinct from those isolated in other countries, likely characterized by separate evolutionary paths [20,43,46,47].

Genetic analyzes of IDV strains circulating in Europe in 2009–2022 showed an increasing diversity resulting from mutations in the HEF glycoprotein (genetic drift), reassortment, or the introduction of strains from the new clade. The nucleotide substitution rate of IDV HEF glycoprotein was significantly higher than HEF of ICV, and no significant differences were found compared to the HA of seasonal human H1N1, H3N2, and IBV [48].

Phylogenetic analyzes were performed for IDV strains circulating mainly in the cattle population but also in breeding pigs. IDV infections in other animals have been reported mainly based on serological evidence, without virus isolation and subsequent genetic analyses. Whereas, in vitro studies explored the tissue tropism of IDV D/OK and D/660 in the respiratory tract of farm and wild animals. IDV binding to acetylated sialic acid receptors in tissues from various animals was examined using a tissue microarray system. All farm animal species expressed host surface receptors for both D/OK and D/660 viruses. In the water buffalo and Asian elephant, D/660 viruses recognize a specific set of acetylated sialic acid residues, whereas D/OK viruses do not bind. Other animal species, such as camels and hedgehogs, are rich in receptors for both D/OK and D/660 [26,49]. Further research on the host preferences for IDVs from particular phylogenetic lineages is needed.

## 4. Clinical Signs, Diagnosis, and Potential Vaccines

Influenza D virus is transmitted through direct contact and aerosol over short distances. Most data on the clinical picture of the disease are related to cattle infections. The possible presence of asymptomatic carriers has also been described in both cattle and other animal species [50]. Studies based on experimental infection in seronegative calves have shown that the infection causes mild to moderate respiratory symptoms, including dry cough, unilateral or bilateral serous/mucous nasal discharge, serous ocular discharge, depression, and short breath. These symptoms result from inflammation and damage to the virus replication sites, i.e., the epithelium of the upper and lower respiratory tract [43,50,51]. The presence of neutrophils has been confirmed in the nasal auricle, trachea, or bronchial lumen [50,51,52]. Clinical signs may vary depending on IDV co-infection, for example, with other pathogens causing bovine respiratory disease (BRD). Experimental infection of pigs showed that IDV replicated in the upper respiratory tract of animals, with detectable virus shedding in nasal swabs, although no clinical signs of infection were observed. Other studies reported that IDV RNA was detected in pig lungs, implying IDV infection also in the lower respiratory tract [53].

Diagnostic samples include nasopharyngeal swabs or lung tissue, as well as animal sera. Direct diagnosis of IDV is based on virus isolation and real-time polymerase chain reaction (PCR) methods [54,55]. Indirect diagnosis of IDV aims to measure serum antibodies against IDV, primarily through virus neutralization (VNT) and hemagglutination inhibition (HI) tests. A competitive ELISA based on monoclonal antibodies has also been developed and validated, but it is not commercially available [56].

There are currently no vaccines against IDV available. At the experimental level, a DNA vaccine expressing the HEF glycoprotein has been demonstrated to be protective against two IDV lines (D/OK and D/660) in guinea pigs [57]. Another study tested an inactivated IDV vaccine in calves. It has been found to be immunogenic and provide partial protection [52]. A recombinant IDV strain (rD/OK-AL) was generated by introducing mutations responsible for the low-temperature adaptation to the PB2 and PB1 proteins using reverse genetics [58]. Results imply that the rD/OK-AL might be a potential candidate for the development of live attenuated vaccines for IDV. Consideration of including IDV in commercial BRD vaccines could improve their effectiveness.

## 5. Host Range of IDV

**Figure 4:**
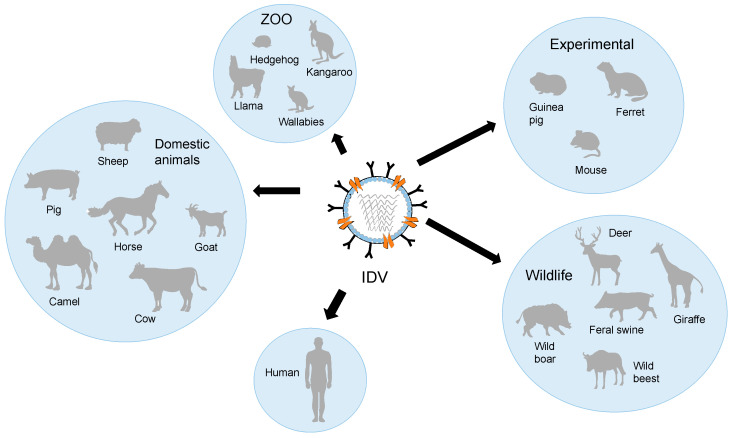
Schematic host range of IDV.

We have discussed the occurrence of IDV in various animal species and presented the findings of experimental studies. A schematic host range for IDV is shown in Figure 4.

### 5.1. Cattle

Cattle are the most extensively tested for IDV. Both serological and virological surveillance confirms the significance of cattle as a reservoir of the virus [12,55]. It is also suggested that IDV plays a significant, potentially initiating role in BRD along with viruses such as bovine herpesvirus-1 (BoHV-1), bovine viral diarrhea virus (BVDV), bovine respiratory syncytial virus (BRSV), and bovine parainfluenza virus type 3 (BPIV3) [51,59,60,61], which is also supported by metagenomic studies [60,62].

In North America, extensive research has been conducted in the USA, repeatedly confirming the circulation of IDV in the cattle population. Studies in Nebraska revealed that all 40 randomly selected farms (2003–2004) had IDV-seropositive adult animals, and approximately 98% of newborn calves had high levels of maternal antibodies to IDV [12]. Silveira et al. conducted a serological study with 1992 bovine serum samples collected across the USA in 2014 and 2015. Seropositive animals were confirmed in 41 US states, revealing a high overall seroprevalence of IDV at an average level of 77.5% [63]. Serological investigations also indicated the presence of antibodies to IDV in cattle in Canada (5.3%) [64]. Metagenomic characterization of viruses associated with BRD has confirmed the prevalence of IDV in animals in Mexico [62]. In Europe, the circulation of IDV in cattle was first reported in France in 2012 and later confirmed in Italy, Denmark, Luxembourg, Ireland, Sweden, Switzerland, and the UK [65,66,67,68,69,70,71,72,73,74]. The highest seroprevalence against IDV in Europe was confirmed in Ireland (94.6%), Italy (92.4%), and Luxembourg (80.2–82.5%) [66,67,68]. Seroprevalence studies of cattle in France have shown a lower frequency—47.2% [19]. It should be noted that in Ireland, Luxembourg, and Italy the pools of tested sera were about 400, while in France the seroprevalence was estimated by testing more than 3000 samples. Molecular studies based on the detection of IDV genetic material in either upper respiratory tract swabs or lung tissues of cows in Europe most often showed a few percent of positive animals, for example, 5.6% in Ireland, 6.5% in Italy, and 8.7% in the UK [66,69,70]. In Switzerland, nasopharyngeal swabs from cows with symptoms of BRD were tested. Based on the analysis of 764 swabs, the following pathogens were found: BRSV, 2.1%, BPI3V, 3.3%, BCoV, 53.5%, and IDV at the level of 4.1% [71]. In Denmark, among 100 calf herds tested, IDV was detected in animals from 12 herds [72], and metagenomic studies showed that IDV was one of the top three viruses associated with BRD in dairy calves [73].

In Sweden, bulk tank milk samples collected during 2019 and 2020 were tested using an in-house indirect ELISA, detecting 32% and 40% of samples as positive for IDV antibodies, respectively [74]. Cow milk testing can provide an alternative in IDV surveillance in a given cow population because dairy cows are kept in herds for long periods. However, seroprevalence results derived from antibody levels in milk may be underestimated because adult cows are usually less susceptible to viral respiratory infections than younger individuals.

In Asia, IDV was first reported in China in 2014, but it is assumed that the virus had been circulating in the cattle population since 2010 [18,43]. Molecular studies in Guangdong province showed that 12.8% of dairy cattle and 7.3% of native yellow cattle were positive for IDV. The seroprevalence was estimated at 7.8% (*n* = 193) for dairy cattle and 5.9% (*n* = 51) for buffaloes [75]. Studies carried out in Japan showed a higher seroprevalence against IDV in cattle than in China. The analysis of bovine sera from Hokkaido (2009–2018) showed a seroprevalence ranging from 45% to 71%, while another study of animals from Japan (2010–2016) showed an average seroprevalence of 30.5% [7,76]. During studies in Hokkaido, three strains of IDV were isolated. The analysis showed the co-occurrence of viruses with different genotypes and antigenic properties in this area [76]. A recent molecular study in the Republic of Korea confirmed the presence of IDV in 1.4% of bovine nasal swabs and lung tissue samples (*n* = 999), while the seroprevalence was estimated at 54.7% (*n* = 742) [77]. In Turkey, molecular studies have also confirmed the presence of IDV; among 76 samples from 12 cattle herds, IDV was detected in 3 cattle in the herd [78].

The seroprevalence of IDV in cattle has been estimated in some African countries, such as Benin at 1.9% (*n* = 201), Togo at 10.4% (*n* = 201), and Morocco at 35% (*n* = 200). Molecular studies have also confirmed the presence of IDV in animals in Namibia [17,41].

The first molecular detection of IDV in South America was in Brazil, with the samples being nasal swabs from a symptomatic cow. Further studies showed that the virus was phylogenetically divergent from known IDVs described in North America, Europe, and Asia [14]. Studies in Argentina indicated that, among 165 tested sera, 68% were positive for IDV antibodies [79]. In Australia, IDV RNA was detected in nasal and nasopharyngeal swabs in symptomatic and asymptomatic animals. Viruses circulating in Australia were grouped within the D/Yama 2016 and D/Yama2019 lineages [15].

Seroprevalences for IDV in cattle range from 1.9% to 94.6%, depending on the region studied. The differences may result from the use of different breeding systems, the frequency of introducing young animals, and the size of the herd. Gaudinoet et al. assume that commercial trade in livestock may play a role in the spread of the virus between European countries and, thus, may be the cause of differences in seroprevalence against IDV. The exposure level of cattle and pigs in destination countries (e.g., Italy) is higher than in the countries of origin (e.g., France) [80].

### 5.2. Swine

Circulation of IDV in the swine population has been confirmed in France, Italy, Ireland, Luxembourg, the USA, Uganda, Korea, and China [53,68,81,82]. Large-scale tests were carried out in France, where, out of over 2000 samples tested, the presence of anti-IDV antibodies was confirmed for 31 pig sera. PCR testing of porcine nasal swabs (*n* = 452) was negative for the virus [53]. In Italy, Foni et al. performed both molecular and serological tests for IDV. Over 800 samples from animals from pig farms affected by respiratory failure were tested, the virus was detected in 2.3% of samples. Genetic analysis showed that viral strains were related to D/swine/Oklahoma/1334/2011. Serological tests conducted on over 3000 animals (2015) indicated a seroprevalence of 11.7%. A study of archival sera from 2009 showed a significantly lower percentage of positive results (0.6%) [81]. Another molecular study confirmed the presence of IDV in pigs in Italy [13,83]. In Luxembourg, IDV seroprevalence in pigs was estimated to range from 0% to 5.9% in 2012–2015 [68]. In Ireland, IDV seroprevalence in pigs was 5.8% (*n* = 377) [67]. IDV-seropositive pigs were also detected in Uganda, where among 166 samples tested, 20.5% were positive [84]. In the USA in 2016, out of nearly 3000 samples tested, IDV was detected in two samples (0.07%) [82]. In turn, screening of pig sera collected in 2011 in the USA (*n* = 220) showed a seroprevalence of 9.5% [21]. Limited molecular testing of samples in Guangdong Province, China, showed positive results in 36.8% of swabs and 28.9% of lung samples [75]. In Korea, more than 2000 porcine nasal swabs and lung tissue samples were tested, and IDV seroprevalence was investigated by testing more than 1600 serum samples. The presence of viral RNA was not confirmed, but the seroprevalence was estimated at 1.4% [77]. Studies indicate that the seroprevalence of IDV in pigs compared to the seroprevalence in cattle is much lower. However, due to the special role of pigs as a mixing vessel in relation to influenza A virus, it should be assumed that the role of pigs in IDV ecology is still an open question.

### 5.3. Camels

Camels from Africa, Asia, and Australia have been tested for IDV. In Kenya, based on HI tests performed on over 290 dromedary camels, seroprevalence was estimated at 99% (geometric mean titer, GMT 150). Testing of the same animals for antibodies to ICV also gave a positive result of 94%, but at lower titers—GMT 38. The authors suggested that further research is warranted to clarify the cross-reactivity of both viruses [17]. Studies of dromedary camels from Ethiopia (Bati *n* = 21 and Fafen *n* = 17) showed IDV seroprevalence of 95.5% and 17.6%, respectively [17]. Subsequent studies of asymptomatic Bactrian camels in Dundgovi, Zavkhan, and Umnugovi provinces in Mongolia (*n* = 40) confirmed the lack of seroprevalence of the animals against IDV [85].

Further testing for the presence of IDV involved archival camel sera, which were originally tested for various pathogens [86,87,88,89,90]. In these studies, very high IDV seroprevalence was observed: 98% in Mongolia (*n* = 100), 100% in Saudi Arabia (*n* = 76), 100% in Australia (*n* = 23), and 100% in Nigeria (*n* = 100). Seroprevalence tests for ICV were also performed, but lower titer values were obtained compared to IDV.

Although a limited number of samples were tested, these studies suggest that camels may play an important role in the circulation of IDV, perhaps acting as a reservoir of IDV alongside cattle.

### 5.4. Small Ruminants

The susceptibility of sheep and goats to IDV infection was confirmed, while the observed seroprevalence is generally lower than in cattle, camels, or pigs. Mazzetto et al. demonstrated that IDV successfully replicates in nasal, tracheal, and lung ovine tissues [91]. Studies conducted in the USA and Canada confirmed the presence of antibodies to IDV in 5.2% (*n* = 557) of sheep, while in the case of the tested goats, 8.8% (*n* = 91) of animals were positive [22]. In France, over 1400 sheep sera and over 600 goat sera from different parts of the country were tested. Studies have shown a seroprevalence of 0.5% for sheep and 3.2% for goats [19]. In Ireland, sheep sera from 2016 and 2017 were tested (*n* = 288), results confirmed seroprevalence against IDV at the level of 4.5% [67]. The highest seroprevalence of goats at the level of 33.8% (*n* = 80) was determined during studies in China [75]. In Togo, Africa, seroprevalence of small ruminants was estimated at 2.2% and 1.4% for sheep and goats, respectively; animal sera were collected between 1991 and 2015 [17]. Subsequent studies conducted in West and East Africa in 2017–2020 confirmed IDV circulation in small ruminants, with seroprevalence at the level of 2% (*n* = 392) and 4.1% (*n* = 417) in sheep and 3.7% (*n* = 163) and 4.4% (*n* = 817) in goats being observed [84].

### 5.5. Horses

A study of 364 sera from horses in the US Midwest in 2015 showed 11–12% seroprevalence against the two predominant IDV lineages (D/OK and D/660) [11]. Studies in the UK showed 0.3% of animals positive in the HI test, while no IDV RNA was detected in equine respiratory samples. Of the additional 430 serum samples initially tested using the pseudotype virus neutralization test (PVNT), 1.4% were considered positive [92]. The results of the study by Sreenivasan et al. on experimental equine infection showed that IDV does not cause respiratory disease in equidae; however, the virus can replicate in the respiratory tract of horses, and the animals seroconvert, suggesting that interspecies transmission of IDV to equidae may occur in nature [93].

### 5.6. Wild Animals

Feral swine may play an important role in the IDV ecology in the USA by serving as a vector between domestic and wild animals (as in the transmission of Brucella suis and IAV) [23,94,95]. Feral swine in the US are escaped domestic animals, imported wild boars, or their hybrids. These animals have spread to about 40 US states, and their number is estimated to be about 5 million individuals. An experimental infection on feral swine showed that IDV was able to replicate in both the upper and lower respiratory tract; viremia was also observed, but no clinical signs of the disease appeared in the infected animals [23]. The seroprevalence of feral swine was studied using two hundred fifty-six sera collected from animals from four US states in 2012–2013 and was estimated at 19.1%. Additionally, a study of 96 archived IAV-seropositive samples showed IDV seroprevalence of 42.7% [23]. In Corse, France, 0.5% of sera from wild boars hunted in 2009–2016 (*n* = 644) tested positive for IDV with low HI titers [53]. It seems that in France only occasional infections in wild boars occur.

Archived sera samples from white-tailed deer (WTD, *n* = 264) from North America were also tested for IDV. According to data from 2018, the number of individuals of this species is approximately 30 million (AFWA, 2018). The results of the HI test showed that 4.9% of the animals were seropositive [24]. The seroprevalence of IDV was also studied in deer cohorts from Europe. In the case of German red deer, roe deer, and fallow deer sera from six national parks were analyzed. Among seropositive animals, there were seven red deer, seven roe deer, and two fallow deer. The overall seroprevalence for IDV was 12% (*n* = 150). In Belgium, a study of archival deer and roe deer sera from 2009–2017 for IDV antibodies showed a seroprevalence of 0.7% (*n* = 283) [90]. In Namibia, IDV RNA was detected in giraffes (*n* = 2) and wildebeest (*n* = 1) [41].

Sera from animals kept in captivity in zoos or wildlife centers in France were also tested for IDV [90]. Studies of hedgehogs have shown that among 48 of sera, 14 were positive for IDV. Among 186 sera from captive fauna, single positive individuals such as kangaroos, wallabies, white-tailed deer, hog deer, and llama were confirmed, with antibody titer ranging from 1:40 to 1:1280.

A summary of serologically positive animals and molecularly confirmed cases of influenza D infections is presented in Table 1. The table contains data regarding both domestic and wild animals. To confirm IDV infection the HI test, ELISA, and real-time PCR were used, next generation sequencing was occasionally performed. IDV-infected animals have been identified in Europe, Africa, Asia, South America, North America, and Australia.

### 5.7. Mice, Guinea Pigs, Ferrets, and Selected Cell Lines

Experimental studies have confirmed susceptibility to IDV infection in mice, guinea pigs, and ferrets [21,93,101,102]. Although the infection in animals was asymptomatic, changes in the lungs were observed in both guinea pigs and mice. In guinea pigs, extensive inflammatory changes, destruction of the bronchial epithelium, and exudates occurred [93]. An increase in the number of neutrophils and lymphocytes in the lungs was observed in mice [101]. Additional transcriptomic analyses showed that IDV replication in mice led to the activation of pro-inflammatory genes, including interferon-gamma (IFN-γ) and the chemokine CCL2 [101]. An in vitro cellular tropism study showed IDV can infect several cell lines such as swine testicle (ST), Madin-Darby canine kidney (MDCK), Green African monkey kidney (Marc-145), human rectal tumor (HRT-18G), and human lung adenocarcinoma (A549) cells [21,103].

## 6. Zoonotic Potential of IDV

The study of human sera in the USA and Canada in 2011 showed an IDV seroprevalence of 1.3%, while a study in Italy showed a seroprevalence ranging from 5.1% in 2005 to 46% in 2014 [21,104]. Leible et al. assessed IDV exposure among thirty-one workers on five large dairy farms; the presence of IDV was found in the nasal washes of 67% of people at least once during the 5-day study period. However, IDV presence was not associated with respiratory symptoms in these workers [27]. Despite serological studies, there is no direct evidence that IDV can infect humans, and no infections in humans have been reported to date. Studies on the properties of IDV receptors [105], the virus replication capacity in a model of human respiratory epithelium [103], as well as the detection of viral genetic material in airport bioaerosol [106], in a hospital [107], and in a pig farmer’s nasal swabs [108] suggest that humans may be susceptible to infection.

## 7. Conclusions

The influenza D virus is unique among other influenza viruses in terms of virion properties as well as host range and reservoir. As with influenza A viruses, a wide host range gives IDV a particular opportunity to reassort, mutate, and promote viral diversity. In most European countries where IDV seroprevalence studies were conducted in cattle, the results indicate several dozen percent of positive animals, and an equally high seroprevalence rate is recorded in some parts of Asia and the USA. Cattle may play an important role in the spread of IDV around the world, including being a source of infection for other farm animals, wild animals, and humans. It should be noted that high seroprevalence is observed also in camels, which may constitute a reservoir of IDV in tropical regions. However, the level of seroprevalence against IDV is much lower in pigs, small ruminants, and horses compared to cattle. Among wild animals, the most extensive research has been conducted on feral swine in the USA and various species of deer in both the USA and Europe. The results confirmed the presence of antibodies against IDV in these animals. Given the abundance of both feral swine in the USA and deer in Europe, these groups may be important in the ecology of the virus. Molecular or serological tests confirmed the occurrence of IDV infection in different animal species: giraffes, kangaroos, wildebeests, wallabies, llamas, and hedgehogs. It is assumed that influenza D may pose a threat as a zoonotic disease, and seroprevalence was confirmed in people with professional contact with cattle. It seems important to monitor the occurrence of IDV not only in domestic animals but also in wild animals, which may act as natural reservoirs. The infectivity and cross-species transmission capacity of IDV make the virus an increasing epidemiological threat, requiring surveillance and further research.

## Figures and Tables

**Table 1:** Summary of serological results and molecularly confirmed cases of influenza D infections, including country, year, animal species, and type of methods used.

Country, Year	Animal Species	Method	References
**EUROPE**			
Belgium (BE), 2019	deer	HI	[90]
Denmark (DK), 2019	bovine	real-time PCR	[72]
France (FR), 2009–2018	swine and wild boar	HI and real-time PCR	[53]
France (FR), 2011–2018	bovine, small ruminants	HI	[19,65]
France (FR), 2019	hedgehogs	HI	[90]
Germany (DE), 2019	deer	HI	[90]
Ireland (IE), 2014–2016	bovine, swine, and sheep	HI and real-time PCR	[69]
Italy (IT), 2012–2019	bovine and swine	PCR and real-time PCR	[13]
Luxembourg (LU), 2012–2016	bovine and swine	HI	[68]
Netherlands (NL), 2021	swine	real-time PCR	[96]
Sweden (SE), 2019	bulk tank milk	ELISA	[74]
Switzerland (CH), 2016	bovine	real-time PCR	[71]
United Kingdoms (UK), 2017	bovine	real-time PCR	[70]
**AFRICA**			
Benin (BJ), 2012–2014	bovine	HI	[17]
Ethiopia (ET), 2019	camels	HI	[85]
Kenya (KE), 2015	camels	HI	[17]
Morocco (MA), 2015	bovine	HI and MN	[17]
Namibia (NA), 2020	wildebeest and giraffe	real-time PCR	[41]
Nigeria (NG), 2021	bovine	real-time PCR	[97]
Nigeria (NG), 2019	camels	HI	[90]
Togo (TG), 2017–2019	bovine	HI	[98]
**ASIA**			
Japan, Yamagata (JP-06), 2019	bovine	real-time PCR	[99]
Japan, Hokkaido (JP-01), 2018	bovine	real-time PCR	[76]
China, Guangdong (CN-44), 2016	bovine, swine, and caprine	real-time PCR	[75]
China, Shandong (CN-37), 2014	bovine	real-time PCR	[18]
Malaysia (MY), 2018	swine	real-time PCR	[28]
Mongolia (MN), 2019	camels	HI	[90]
Republic of Korea (ROK), 2019	bovine and swine	HI and real-time PCR	[77]
Saudi Arabia (SA), 2019	camels	HI	[90]
Turkey (TR), 2018	bovine	Real-time PCR	[78]
**SOUTH AMERICA**			
Argentina (ARG), 2013	bovine	ELISA	[79]
Brazil (BR), 2020	bovine	real-time PCR	[14]
**NORTH AMERICA**			
USA, California (CA), 2018	bovine	real-time PCR	[44]
USA, Hawaii (HI), North Carolina (NC), Oklahoma (OK), Texas (TX), 2012–2013	feral swine	HI	[23]
USA, Kansas (KS), 2010–2012	bovine	real-time PCR	[20]
USA, Kentucky (KY), 2017	swine	next generation sequencing	[100]
USA (samples collected across the country)/2014, 2015	bovine	HI	[60]
USA, Mississippi (MS), Michigan (MI), Minnesota (MN), and Oklahoma (OK), 2011–2017	deer	HI	[24]
USA, Mississippi (MS), 2004–2014	bovine	real-time PCR, HI	[55]
USA, Nebraska (NE), 2003–2004, 2012–2014	bovine	HI	[12]
USA, Oklahoma (OK), 2011–2013	swine	next generation sequencing	[21]
USA, 12 states, 2014	bovine	real-time PCR	[16]
Canada, Quebec (QC), 2020	bovine	real-time PCR	[64]
Mexico (MX), 2015	bovine	real-time PCR	[62]
**AUSTRALIA**			
Australia—New South Wales (AU), 2019	bovine	transcriptomics	[15]
Australia (AU), 2019	camels	HI	[90]
